# Fungal Sinusitis Spreading to the Sellar Region Mimicking a Pituitary Tumor: Case Report and Literature Review

**DOI:** 10.3390/jof11030233

**Published:** 2025-03-19

**Authors:** Sandra Pekic Djurdjevic, Valentina Arsic Arsenijevic

**Affiliations:** 1 Neuroendocrine Unit, Clinic for Endocrinology, Diabetes and Diseases of Metabolism, University Clinical Center, 11000 Belgrade, Serbia; sanendo1@gmail.com; 2 School of Medicine, University of Belgrade, 11000 Belgrade, Serbia; 3 National Medical Mycology Reference Laboratory (NMMRL), Institute of Microbiology and Immunology, 11000 Belgrade, Serbia

**Keywords:** sinusitis, fungi, sella turcica, pituitary gland, fungus ball, bone destruction

## Abstract

Chronic fungal sinusitis (FS) can cause bone erosion and extend to the sellar region, often misdiagnosed as pituitary tumors or malignancies. We report a 56-year-old immunocompetent female with sphenoid FS presenting as a giant sellar mass compressing the optic chiasm, with normal pituitary function. The surgery successfully resolved her symptoms, and a histological examination confirmed the presence of a fungal hyphal mass. We conducted a literature review of 52 publications on FS cases with bone erosion and inflammatory extension to the sellar region, which included analyses of 67 patients (35 females, mean age 49.6 years, half immunocompetent). The most common symptom was headache (73.1%), followed by visual complaints (71.7%), visual deterioration (40.3%), ophthalmoplegia (38.8%), and visual field defects (13.4%). Symptom duration averaged 4.5 months in 65.7% of cases. *Aspergillus* was the most frequent (71.6%). Hormonal abnormalities included hypopituitarism (25.4%) and hyperprolactinemia (13.4%). Surgery was performed in 92.5% of patients. Common diagnoses included pituitary abscess (41.8%), fungal granuloma (16.4%), aspergillosis (16.4%) and allergic FS (14.9%). Antifungal therapy was administered in 53.7% of cases. Cure was achieved in 67.2%, while the mortality rate was 10.4%. Early recognition of fungal involvement, supported by a multidisciplinary approach, is essential for the accurate diagnosis and effective treatment. This highlights the need for vigilance to improve the outcomes in similar cases.

## 1. Introduction

A variety of fungal organisms, exhibiting geographic variations in frequency, have been identified within nasal mucin in patients with acute or chronic sinusitis, as well as in healthy individuals without sinusitis [1,2,3]. In contrast to invasive fungal sinusitis (FS)—an acute, life-threatening condition primarily affecting highly immunocompromised patients—fungal ball (sinus mycetoma) and allergic fungal sinusitis (AFS) are chronic, indolent conditions occurring in immunocompetent individuals [1,4,5,6,7,8,9]. These non-invasive forms of FS generally have a favorable prognosis, as the fungus ball typically consists of well-organized mycelium without tissue invasion. They are characterized by the absence of fungal hyphae within the mucosa, submucosa, bone, or blood vessels of the paranasal sinuses.

Epidemiological studies in Serbia have revealed that fungal diseases affect approximately 16.5% of the population. Within that, 14.3% suffer from chronic or allergic fungal diseases, which impact quality of life, while 2.2% experience life-threatening fungal infections [10].

Although rare, non-invasive FS can progress to invasive fungal sinusitis. AFS arises from a poorly controlled inflammatory and immunological response to fungal hyphae colonizing the sinuses [11,12,13,14,15]. Although AFS is widely considered a non-invasive pathological process, bony erosion can occur in certain cases. When associated with granulomas, the disease tends to exhibit more aggressive behavior. Patients with granuloma-associated AFS show higher rates of orbital erosion, skull base involvement, proptosis, facial pain, and recurrent surgeries compared to those without granulomas [16].

Longstanding untreated cases of AFS or fungus ball can lead to bony changes, erosion, and direct spread to critical regions such as the sellar region, cavernous sinuses, or orbital apex [15,17,18,19,20,21,22,23,24,25,26,27,28,29,30,31,32,33,34,35,36,37,38,39,40,41,42,43,44,45,46,47,48,49,50,51,52,53,54,55,56,57,58,59,60,61,62,63,64,65,66,67,68,69,70,71,72,73,74,75,76]. The simultaneous involvement of these areas is referred to as cavernous sinus/orbital apex syndrome. In severe cases, an invasive form of fungal infection may develop in high-risk patients, potentially leading to fatal outcomes.

In some patients, sinus mucoceles may arise from chronic immune reactions, typically affecting the frontal or ethmoid sinuses unilaterally. These mases can exert pressure on the bone, potentially causing skull base erosion and intracranial extension. This can result in complications such as cerebrospinal fluid leakage, brain herniation, or infection. Such cases are often misdiagnosed as pituitary tumors or sellar abscesses due to their nonspecific clinical and radiological presentation [71].

An inflammatory sellar pseudotumor, characterized by infiltration of acute and chronic inflammatory cells with variable fibrous responses, may arise from the spread of bacterial or fungal sinusitis. This can involve soft tissue infiltration with or without bone erosion [77]. MRI is superior to CT for assessing extrasinonasal extension, with typical findings including hypointensity or isointensity on T1-weighted images and hypointensity on T2-weighted images with fat suppression [77].

We previously reported a challenging case of an immunocompetent male with longstanding aggressive AFS caused by *Schizophyllum commune* [45]. This case involved sellar bone erosion and eosinophilic inflammation extending to the sellar and suprasellar regions, mimicking a giant pituitary tumor. The patient presented with hyperprolactinemia due to pituitary stalk compression. Despite two unsuccessful surgeries, he achieved complete recovery following combined treatment with systemic antifungals, corticosteroids, and a third surgery.

Few systematic reviews and case series on fungal propagation to the sellar region, cavernous sinuses, and orbits have been published over the past decades. These studies describe clinical presentations, risk factors, and imaging findings [28,52,71].

In this case, in the presentation we focus specifically on our challenging case of sphenoid fungal sinusitis extending to the sellar region, forming a pituitary pseudotumor, along with a literature review to compare the clinical presentation, risk factors, laboratory findings, fungal types, radiological appearances, pathohistological findings, treatment approaches, and outcomes, with a particular emphasis on hormonal evaluation, given the critical endocrine role of the pituitary gland. 

## 2. Materials and Methods

We present a case of fungal sinusitis with bone invasion extending to the sellar region, detailing the clinical presentation, hormonal analysis, MRI findings, treatment approach and clinical outcome. Then, we conducted a literature review, providing a comprehensive overview of the existing knowledge on the invasion of FS through bone to the brain and sellar region, using the Medline database (PubMed). The following English keywords were used for the search: sinusitis, fungal-fungi, sella turcica, pituitary gland, invasive, bone destruction. In addition to electronic searches, manual journal reviews were conducted, and relevant cross-references from identified articles were included.

Articles published between 1969 and October 2024 were reviewed. Inclusion criteria were original case reports and small studies on sphenoid FS with bone erosion and extension to the sellar region, written in English. Studies involving in vitro or animal models, review articles without original case reports, and studies unrelated to the topic were excluded.

Two authors (SPD, VAA) independently screened the titles and abstracts of all identified articles to select the potential case reports and small series focusing on fungal pituitary abscess. Full-text articles were reviewed based on the inclusion and exclusion criteria to ensure appropriateness for inclusion. Data extraction was conducted independently by the same two evaluators, with disagreements resolved through consensus. The extracted data included: demographic details (age, sex, ethnicity), presenting symptoms, signs, and their duration prior to diagnosis, evidence of bone erosion and sphenoid sinusitis on CT or MRI, type of fungal sinus disease, treatment modalities, identified fungal pathogens, additional antifungal therapies, and clinical outcomes.

The search yielded 52 articles in PubMed/MEDLINE, comprising 45 case reports and 7 small studies (each involving 2–5 patients).

## 3. Results

### 3.1. Case Presentation

**Figure 1:**
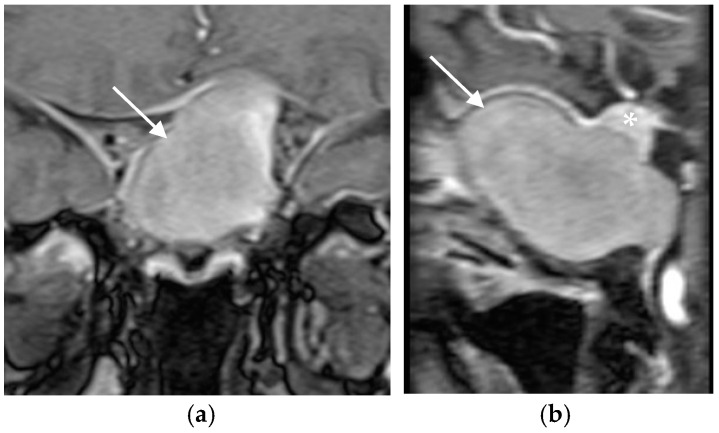
(**a**) Coronal and (**b**) sagital MRI scans of the sellar region before the transphenoidal operation showing giant hyperintense (on T_1_-weighted sequences) lesion in the sellar region (white arrows), with a thin rim of enhancement propagating in all directions (supra-, infra-, retro- and pre-sellar). The pituitary gland was displaced superiorly (asterix).

A 56-year-old immunocompetent female with no significant medical history presented with nasal congestion, headache, visual disturbances, and bitemporal hemianopia. Sellar magnetic resonance imaging (MRI) revealed a giant mass in the sphenoid sinus and clival region, accompanied by bone destruction and extension into the sellar region and surrounding structures (supra-, infra-, retro-, and pre-sellar regions; Figure 1a,b). The pituitary gland was displaced superiorly, prompting referral to a neurosurgeon and endocrinologist.

The patient had no history of allergies, diabetes, malignancies, intravenous drug use, or blood transfusions. She was a nonsmoker and did not consume alcohol or use illicit drugs.

### 3.2. Clinical Examination and Laboratory Findings

On examination, the patient was alert, oriented, afebrile, and had normal vital signs. Nasal congestion was present without discharge. Neurological evaluation revealed no cranial nerve palsies but confirmed a visual field defect consistent with bitemporal hemianopia.

Routine laboratory investigations, including complete blood count, sedimentation rate, fibrinogen, C-reactive protein, blood glucose, electrolytes, liver enzymes, and immunoglobulins, were all within normal limits. Imaging studies, including chest radiography, thyroid, and abdominal ultrasonography, showed no abnormalities. Skull radiographs demonstrated an enlarged sella with erosion of the dorsum.

Endocrinological assessment revealed no signs of arginine vasopressine deficiency (or diabetes insipidus). Basal hormonal levels measured at 08:00 AM after an overnight fast, including FT4, TSH, gonadotropins, ACTH, cortisol, prolactin, and IGF-I, were within normal ranges.

The radiological and clinical findings strongly suggested a sellar mass lesion with a suprasellar extension, mimicking a giant pituitary adenoma.

### 3.3. Surgical and Pathological Findings

**Figure 2:**
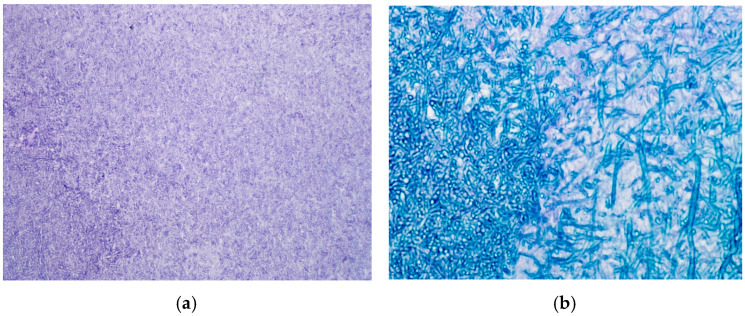
(**a**) The sellar content obtained during the transphenoidal operation stained by Haematoxylin and Eosin (H&E × 250). (**b**) The fungal hyphae within the sellar content (Grocott Methenamine Silver × 250) confirmed the fungal etiology.

The patient underwent transsphenoidal debridement of the lesion. The material was removed from the sella, and bone erosion was noted during the procedure. Histopathological examination of formalin-fixed, paraffin-embedded tissue stained with hematoxylin and eosin (H&E) confirmed the presence of fungi (Figure 2a). Grocott Methenamine Silver staining revealed fungal hyphae (Figure 2b). There was no evidence of fungal tissue invasion, pituitary adenoma, or other pre-existing pituitary lesions.

The surgically removed pathological sellar content was identified as a mass of hyphae or fungus ball and was excised in its entirety. There were no macroscopic or microscopic signs of angio-invasion or tissue invasion. The sellar content was well-localized and completely removed. Preoperatively, neither the neurosurgeon, endocrinologist, nor radiologist suspected a fungal infection (e.g., fungus ball). Consequently, laboratory investigations for markers such as galactomannan or fungal glucans were not performed. Intraoperatively, there were no signs suggestive of a fungal or bacterial infection, so the material removed from the sellar region was not sent for microbiological analysis and cultivation. Postoperatively, we analyzed material obtained from the nasal cavity and blood. Three consecutive samples were collected from the nasal cavity and cultured on standard mycology media, including Sabouraud Dextrose Agar and Potato Dextrose Agar plates (Promedia, Kikinda, Serbia). The plates were incubated at two different temperatures, 26 °C and 37 °C, in parallel. Additionally, we tested blood sera for *Aspergillus* IgG and *Aspergillus* IgM antibodies using the Platelia assay (Bio-Rad, Hercules, CA, USA). However, mycological results remained negative throughout the seven-day cultivation period, as did serological testing. Additional laboratory diagnostic options were not available.

### 3.4. Outcome

**Figure 3:**
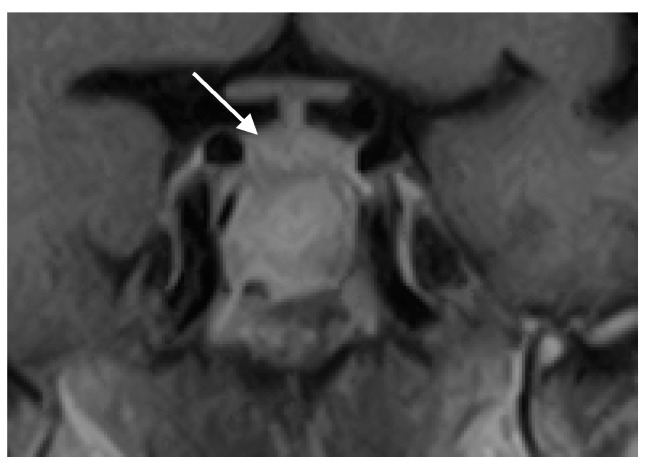
Coronal MRI scan of the sellar region after the operation showing a complete resolution of the pathological process and a normal pituitary gland (marked with white arrow).

Postoperatively, the patient experienced significant improvement in headache and normalization of vision and visual fields. The patient in this case was treated exclusively with surgery, without a postoperative antifungal therapy. She was cured by surgery alone. Follow-up examinations at three months and one year post-surgery demonstrated a complete resolution of symptoms without disease recurrence (Figure 3). Pituitary function remained intact, and no additional therapy was required. The patient was followed for three years postoperatively with annual MRI scans, which showed no signs of FS or pituitary lesions.

### 3.5. Literature Review

A literature review was conducted to analyze cases of FS invading through bone to the brain and sellar region. A total of 52 publications were identified: 45 case reports and 7 small studies involving 2–5 patients each. In total, data from sixty-seven patients (thirty-five female, thirty male, two unspecified), including one previously reported, and one presented in this publication, were analyzed. The mean patient age was 49.6 ± 2.3 years (range, 17–90).

The most common country of origin was the United States (n = 20, 29.9%), followed by China (n = 16, 23.9%), India (n = 6, 9.0%), and Japan (n = 3, 4.5%). Two patients each (3.0%) were reported from Australia, Germany, Korea, Poland, Serbia and Turkey. Single cases (1.5%) were reported from Morocco, Spain, Iran, Sudan, Mexico, Italy, Malaysia, Côte d’Ivoire, Portugal, and one unspecified location.

### 3.6. Clinical Presentation

The presenting symptoms and signs are summarized in Table 1. The most common symptom was headache (n = 49; 73.1%), followed by at least one visual complaint (n = 48; 71.7%): visual deterioration in 27 patients (40.3%), ophthalmoplegia in 26 patients (38.8%), and visual field defects in 9 patients (13.4%). Less frequent symptoms included hypogonadism (n = 6; 9.0%), dizziness (n = 5; 7.5%), and galactorrhea, proptosis, eye pain, or hemiparesis (n = 2 each; 3.0%). One patient each presented with agitation, altered sensorium, gynecomastia, syncope, facial pain, ear pain, seizures, and hyponatremia.

Symptoms and signs were reported to last for a mean duration of 4.5 ± 0.9 months (range, 0.5–36 months) in 44 patients (65.7%). In five patients (7.5%), symptoms were present for 5.6 ± 1.0 days (range, 2–7 days). For 18 patients, the duration was not reported.

### 3.7. Patient Characteristics and Imaging Findings

Half of the patients (n = 33; 49.2%) were immunocompetent, twelve patients (17.9%) had diabetes mellitus, and seven (10.4%) were immunodeficient. Immunocompetence status was not reported for 15 patients (22.4%).

A sellar mass was identified in all cases. Sphenoid sinusitis was the most common site of infection (n = 48; 71.6%). Among these, two patients had combined sinusitis of the sphenoid, maxillary, and ethmoid sinuses, and three had combined sphenoid and ethmoid sinusitis. Seven patients (10.4%) showed no signs of sinusitis, while data on sinusitis were unavailable for twelve patients (17.9%). Bone erosion was reported in 40 patients (59.7%) but was absent in 5 patients (7.5%); no data on bone erosion were available for 22 patients (32.8%).

### 3.8. Fungal Pathogens, Endocrine Function and Treatment

The most frequently isolated fungus was *Aspergillus* (n = 48; 71.6%). Other identified fungi included *Candida* species (*C. albicans* and *C. glabrata*) in five patients (7.5%), *Schizophyllum*, *Curvularia*, *Mucorales* (*Zygomycetes*), and *Coccidioides* in one patient each (1.5%). In 10 patients (14.9%), the type of fungus was not reported.

Hypopituitarism was present in 17 patients (25.4%; Table 2), while hyperprolactinemia was observed in 9 patients (13.4%). Normal pituitary function was documented in 10 patients (14.9%). Deficiency of arginine vasopressin (AVP) was reported in one patient (1.5%). Three patients had pre-existing pituitary lesions: one with a growth hormone-secreting adenoma (acromegaly), one with an ACTH-secreting adenoma (Cushing’s disease) and one with nonfunctioning pituitary adenoma. In the fourth patient the ACTH level was increased, with no further information about possible ACTH secreting pituitary adenoma. Endocrine function was not reported for 30 patients (44.8%).

Surgical treatment was performed in 62 patients (92.5%). One patient (1.5%) was not operated on, and surgical data were unavailable for four patients (6.0%). The most common pathological and radiological diagnosis (Table 2) was pituitary abscess (n = 28; 41.8%), followed by fungal granuloma or fungus ball (n = 11; 16.4%), aspergillosis (n = 11; 16.4%) and AFS (n = 10; 14.9%). Antifungal drugs were used in 36 patients (53.7%), with amphotericin B and voriconazole being the most common agents (n = 17 each). Other treatments included itraconazole (n = 5), fluconazole (n = 4) and caspofungin (n = 2).

Seven patients with *Aspergillus* sinus infection were treated with amphotericin B; six recovered, while outcome data were unavailable for one patient. Amphotericin B and fluconazole were used in two patients with *Aspergillus fumigatus* sinus infection—one experienced near-total resolution, while the other died. Amphotericin B and itraconazole were administered to three patients (two with *Aspergillus* sinus infection and one with *Schizophyllum* sinus infection), whom all recovered. Amphotericin B and voriconazole were used in three patients with *Aspergillus* sinus infection; two showed partial recovery, while one died. Amphotericin B and 5-fluorocytosine were given to two patients with *Aspergillus* sinus infection—one recovered, and the other showed partial recovery.

Voriconazole was used in eleven patients (ten with *Aspergillus* sinus infection and one with *Candida glabrata* sinus infection). Among them, seven recovered, two showed a reduction in *Aspergillus* granuloma, one died due to stroke, and one was lost during the follow-up. Two patients with *Aspergillus* sinus infection were treated with voriconazole and caspofungin, and both recovered. One patient with *Candida albicans* and *Candida glabrata* sinus infection was treated with voriconazole and fluconazole but did not survive.

Itraconazole was administered to two patients with *Aspergillus* sinus infection, both of whom recovered. Ketoconazole was used in one patient with *Coccidioides immitis* sinus granuloma, who recovered. Fluconazole was given to one patient with *Aspergillus* sinus infection, and he recovered.

### 3.9. Outcomes

Therapy outcomes were reported for 61 patients (91.0%, Table 2). Most patients achieved full recovery (n = 45; 67.2%), while six patients (9.0%) experienced disease regression. Recurrence occurred in two patients (3.0%), and blindness persisted in one patient (1.5%). Seven patients (10.4%) died.

## 4. Discussion

Pituitary infection is a rare condition, accounting for less than 1% of all pituitary lesions [58,78,79,80]. Its etiology includes bacterial, viral, fungal, and parasitic origins, often linked to hematogenous dissemination in immunocompromised individuals, iatrogenic causes following transsphenoidal surgery, or direct spread from adjacent structures such as the meninges, sphenoid sinus, cavernous sinus, or skull base. A systematic review of 488 pituitary abscess cases across 218 studies revealed that over half (54.8%) were culture-negative, with fungal organisms (commonly *Aspergillus*) identified in 8.8% of cases [81].

Fungal sinus diseases, once considered rare, have become increasingly reported over the last two decades [1,3,8,9,82]. Possible reasons for this rise include greater awareness, improved diagnostic methods, and higher prevalence of immunosuppressive conditions such as diabetes mellitus, cancer therapies, HIV, post-transplant therapies, and antibiotic overuse.

The most frequently encountered fungi in clinical practice include *Aspergillus*, *Alternaria*, *Fusarium*, and *Schizophyllum* species. Specifically, *Aspergillus fumigatus* and *Aspergillus flavus* are the main pathogens associated with FS, characterized by hyaline, septate hyphae with acute-angle branching. These species thrive in warm, tropical climates, contributing to their predominance in regions like India and Southeast Asia.

Fungal sinus diseases represent a spectrum of conditions classified as invasive (fungal hyphae invades the tissue through the epithelium) or non-invasive and further categorized as acute (lasting less than four weeks) or chronic (present for at least 12 weeks) [5,6]. Non-invasive FS is more common in immunocompetent patients and is classified as either a fungus ball or AFS [7,8,83].

A sinus fungus ball is an agglomeration of fungal hyphae within the paranasal sinuses. In contrast, AFS is a non-infectious chronic condition characterized by an exaggerated immunological response (eosinophilic inflammation and mucin) to colonizing fungi [84,85]. The first reports of AFS, involving nasal polyposis and positive sinus cultures for *Aspergillus* species, were documented in 1983 [83]. Various fungal organisms, including *Aspergillus*, *Curvularia*, *Penicillium*, *Alternaria*, *Schizophyllum* and *Fusarium*, have been identified in the allergic mucin of AFS patients [86]. AFS is explained as a hypersensitivity reaction to ubiquitous fungal allergens [7,12,13,84] and is diagnosed in more than half of chronic sinusitis cases [13,87]. The underdiagnosis of AFS stems from the difficulty in identifying sparse fungal hyphae in allergic mucin. Diagnosing FS, including AFS, is challenging due to its slow, oligo-symptomatic progression. Symptoms, signs, and radiologic appearances are non-specific, requiring fungal identification for confirmation. Culture and microscopic examination remain the gold standard for diagnosing fungal infections [3]. Although fungi pose significant challenges, they are often overlooked as a potential cause. Obtaining a representative sample for fungal detection can be difficult, and traditional techniques have low sensitivity.

Although fungal ball and AFS are typically non-invasive chronic conditions, they can occasionally spread to adjacent tissues, resulting in more aggressive disease manifestations [15,16,38,45,56,86,87,88,89,90]. Rare complications include bone erosion and fungal spread to the orbital, sellar, or intracranial regions, forming inflammatory pituitary pseudotumors [15,38,45,67,86,88,89,90].

These aggressive forms may compress and displace the pituitary gland, mimicking the effects of pituitary tumors. These patients represent the greatest challenge for diagnosis and treatment. A large prospective study of 251 chronic sinusitis patients found orbital extension in 16.7% and intracranial extension in 6.0% [86]. Our first published case featured a patient with a large intra- and suprasellar mass requiring transsphenoidal and functional endoscopic sinus surgeries for diagnostic and therapeutic purposes [45]. In this case, a 44-year-old immunocompetent man with AFS lasting for seven years due to *Schizophyllum commune* exhibited bony erosion and sellar propagation, presenting with headaches, diplopia, and hyperprolactinemia. Diplopia was transient, caused by cranial nerve compression from inflammatory tissue. Treatment included surgery, systemic and inhaled corticosteroids, and antifungals (amphotericin B and itraconazole).

In this publication we presented our second case of aggressive sphenoid FS in an immunocompetent female, complicated by bone erosion and sellar propagation. The sphenoid sinus’s reduced aeration and anaerobic conditions predispose it to fungal infection. Our literature review shows that sphenoid sinusitis is the most prevalent site of fungal infection with sellar propagation, diagnosed in 71.6% of cases. Patients’ average age was 49.6 years (range: 17–90), with no gender differences observed. Half of the patients were immunocompetent, while others had diabetes mellitus (17.9%) or immunodeficiency (10.4%).

In the literature review, the patients with fungal infections extending to the sellar region presented with mass effects similar to pituitary tumors or abscesses, including headache (73.1%) and at least one visual complaint (71.7%), including visual deterioration (40.3%), ophthalmoplegia (38.8%), and visual field defects (13.4%). Visual field defects are due to optic chiasm compression, while ophthalmoplegia is due to compression of cranial nerves III, IV and VI in cavernous sinuses (with ptosis and diplopia). Symptoms often develop over months (mean: 4.5 months). Unlike bacterial abscesses, these patients typically lack fever, meningism, and leukocytosis. *Aspergillus*, the most common fungus, is angioinvasive and may cause vasculitis, cerebrovascular insult, cavernous sinus thrombosis, or carotid-cavernous fistula, which can be fatal.

Hypopituitarism is the most frequent endocrine complication, particularly hypocorticism and hypogonadism. Hyperprolactinemia, seen in 13.4% of cases in this systematic review, may result from pituitary stalk compression and dopamine insufficiency or fungal glucans’ stimulation of prolactin release [45,91]. In our patient, prolactin level did not normalize after surgical pituitary stalk decompression, but normalized after 4 months of systemic antifungal and corticosteroid therapy for AFS caused by *Schizophyllum commune* [45]. Rarely, AVP deficiency and hyponatremia (due to a syndrome of inappropriate secretion of antidiuretic hormone, SIADH) are observed [92]. In this review, pituitary function was reported in 55.2% of patients, with hypopituitarism present in 25.4% of patients. Hyperprolactinemia was detected in 13.4% of patients. AVP deficiency was present in only one patient, as well as hyponatremia. Pituitary function was normal in 14.9% of patients.

Distinguishing FS with propagation to the sellar region from a pituitary adenoma or a bacterial abscess can be challenging. CT imaging often reveals hyperintense lesions, while MRI findings typically show isointense or hypointense masses on T1-weighted images, with peripheral rim enhancement, calcifications, and low signal intensity due to iron deposits on T2-weighted images. The presence of low signal intensity on T2-weighted images, caused by iron deposits and sinusitis, may raise suspicion for a pituitary abscess [67,81]. Notably, iron serves as an essential growth medium for fungal hyphae. CT imaging excels in detecting bony erosion and hyperdense fungal lesions, whereas MRI is more effective for assessing soft tissue and mucosal involvement [93]. In this review, bone erosion was detected in 59.7% of patients, while 7.5% showed no signs of bone erosion. For the remaining patients, no data on bone erosion were available.

The most frequent radiological and pathological finding in our review was pituitary abscess (41.8%), followed by fungal granuloma (16.4%), aspergillosis (16.4%), and AFS (14.9%). Pituitary abscesses are categorized as primary (arising in an otherwise normal pituitary gland) or secondary (occurring in a pre-existing pituitary mass such as a Rathke’s cleft cyst, craniopharyngioma, or pituitary adenoma) [58]. In our analysis, we identified two patients with functioning pituitary adenomas (acromegaly and Cushing’s disease) and one with nonfunctioning pituitary adenoma. A fourth patient exhibited elevated ACTH levels, although no additional information was available regarding a possible ACTH-secreting adenoma.

A definitive diagnosis requires histopathology and special stains for fungal visualization—Grocott or Gomori methenamine silver staining, fungal culture, and PCR analysis. Fungal serological markers (e.g., 1,3-β-D-glucan, galactomannan) may aid in diagnosis. In this review, *Aspergillus* was the most commonly identified fungus, detected in 71.6% of patients. *Candida* species (*Candida albicans* and *Candida glabrata*) were isolated in 7.5% of patients, while *Schizophyllum*, *Curvularia*, *Mucor*, or *Coccidioides* were identified in 6.0%. Fungal type data were unavailable for 15.9% of patients.

Surgical debridement of infected tissues and sinus drainage is essential for diagnosis, cure, and recurrence prevention [12]. In our review, 92.5% of patients underwent surgery, achieving a cure rate of 67.2%. In our patient, neither the neurosurgeon, endocrinologist, nor radiologist suspected a fungal infection (e.g., fungus ball). The sellar content was well-localized and completely removed, and was identified by a pathologist as a mass of hyphy or fungus ball, with no macroscopic or microscopic sings of tissue invasion or angioinvastion. Following the operation and the pathological confirmation of fungal etiology, we analyzed material obtained from the nasal cavity for fungi; however, mycology results were negative. Surgery remains the treatment of choice for a fungus ball, as it effectively removes fungal mass from the affected place. The surgical intervention successfully cured our patient, who was subsequently monitored for three years without any signs of disease recurrence.

Surgical treatment remains the method of choice for managing a fungus ball, as no definitive guidelines exist regarding the use of systemic antifungal agents in these cases. This might explain why, in our literature review, systemic antifungal drugs were administered to only half of the patients (53.7%), with amphotericin B and voriconazole being the most commonly used agents. In aggressive cases of AFS, long-term antifungal therapy combined with corticosteroids may be necessary. However, the limited drug accessibility to the sellar region should be taken into consideration.

Approximately 15% of patients in this literature review were diagnosed with AFS. Aggressive AFS with sellar propagation poses significant diagnostic and therapeutic challenges. Early recognition and multidisciplinary management are critical to improve outcomes in affected patients. Even decades after the first description of AFS, significant uncertainty remains regarding the optimal management of this condition [94,95]. Systemic antifungals may be considered for patients with frequent recurrences following surgical debridement or in cases with histological evidence of severe pressure erosion [96,97]. The use of topical or systemic antifungals, such as amphotericin B, itraconazole, or fluconazole, has been proposed in the treatment of AFS [98,99,100].

Amphotericin B has been a cornerstone antifungal agent for decades, demonstrating effectiveness against most yeasts and molds. As a polyene antibiotic, it disrupts fungal cell wall integrity by binding to ergosterols, leading to increased permeability, potassium leakage, and cell death [101]. However, conventional amphotericin B in its deoxycholate formulation is associated with significant renal toxicity and infusion-related side effects such as chills, fever, nausea, and hypotension. Lipid formulations of the drug can reduce these toxicities [102].

Itraconazole, a triazole antifungal, inhibits lanosterol 14α-demethylase, a cytochrome P-450-dependent enzyme essential for ergosterol production. This highly lipophilic drug achieves substantial penetration into the central nervous system. In this review, more than half of the patients with fungal sphenoid sinusitis propagating to the sellar region were treated with antifungal drugs (53.7%), predominantly amphotericin B and voriconazole.

A large study involving 67 patients with AFS, treated over eight years with or without a postoperative oral corticosteroid protocol, adopted a treatment approach inspired by strategies used for allergic bronchopulmonary aspergillosis, a related lower respiratory tract condition [103]. Patients received oral prednisone at 0.5 mg/kg every morning for two weeks, followed by the same dose every other morning for two additional weeks. The dose was gradually tapered to 5–7.5 mg every other morning by three months and maintained at 5 mg every other morning for up to a year. Significant clinical improvement was noted after at least two months of postoperative corticosteroid use, with the best outcomes observed after a full year of treatment [103].

Topical and oral nasal corticosteroids also play a critical role in reducing AFS recurrence rates, alleviating recurrent rhinosinusitis symptoms, and controlling the regrowth of inflammatory nasal polyps. While the prognosis for AFS is generally favorable, invasive FS remains a life-threatening condition with a poor prognosis. No definitive guidelines exist regarding the optimal antifungal agents or treatment duration [104]. Our literature review of patients with sphenoid FS with bone erosion and sellar propagation revealed that 67.2% of patients were cured following therapy, 9.0% experienced regression, and 3.0% faced recurrence. Unfortunately, 10.4% of patients succumbed to the condition.

Our study has some limitations. The identification of filamentous fungi was not performed because the patient had no prior clinical indication of a fungal infection in the sinus region extending into the sellar region. Additionally, the surgical sample from the sellar region was obtained solely for histopathological examination and was not submitted for microbiological testing. Nevertheless, with this case, we aim to highlight the need for evolving clinical practices, as the local epidemiology of fungal diseases may play a crucial role in future diagnostic and treatment approaches.

### Future Research Directions

Chronic FS, whether allergic or involving as a fungus ball, may result in bone erosion and intracranial, orbital, or maxillar sinus propagation, leading to severe complications and poses a significant diagnostic and therapeutic challenge. Timely suspicion of fungal etiology is crucial for accurate diagnosis and treatment, particularly in patients with chronic sinusitis who develop headaches and visual issues, such as visual deterioration, ophthalmoplegia, and visual field defects. Therefore, a multidisciplinary approach involving ENT specialists, neurosurgeons, radiologists, pathologists, microbiologists, ophthalmologist and endocrinologists is essential.

Future research should include a systematic review of patients with fungal sinusitis, particularly those with involvement extending to the sellar region. Such a review would be valuable for enhancing our understanding of this condition, improving diagnostic procedures, optimizing therapy, and ultimately improving patient outcomes.

To facilitate the timely detection of this often-neglected condition, we propose integrating specialized questions into a comprehensive platform with specialized fungal-oriented software version 1.0. For future research, we plan to investigate this questionnaire in the Serbian population and apply two different analytic models (multivariate analysis and classification and regression tree analysis) to develop clinical decision rules. The reference standard has identified low, moderate, and high risk ranges for chronic fungal sinusitis. However, these risk ranges are still under development. This research will facilitate a multidisciplinary approach and help medical professionals more effectively identify patients at risk for these complications (Table 3, available on www.fcf.org.rs). However, prospective validation in a different population will be necessary.

## 5. Conclusions

There is no clear professional consensus on the diagnosis and treatment of patients with FS, particularly in cases of aggressive forms that involve bone erosion and extension to the sella region, leading to the formation of a pseudotumor mass. Case presentations and a thorough analysis of published cases are crucial for developing future guidelines for these patients. Until such guidelines are established, surgery remains the gold standard for managing fungus balls. A multidisciplinary approach is essential for both diagnostic and therapeutic interventions, involving ENT specialists, neurosurgeons, radiologists, pathologists, microbiologists, ophthalmologists, and endocrinologists.

## Figures and Tables

**Table 1:** Symptoms and signs of fungal sinusitis with propagation to the sellar region (patients; n = 67).

Symptoms and Signs	n	%
Headache	49	73.1
Visual deterioration	27	40.3
Ophthalmoplegia (cranial nerve palsy)	26	38.8
Visual field defects	9	13.4
Hypogonadism	6	9.0
Dizziness	5	7.5
Galactorrhoea	2	3.0
Proptosis	2	3.0
Eye pain	2	3.0
Hemiparesis	2	3.0
Agitation	1	1.5
Altered sensorium	1	1.5
Gynecomastia	1	1.5
Syncope	1	1.5
Face pain	1	1.5
Ear pain	1	1.5
Seizures	1	1.5
Hyponatremia	1	1.5

**Table 2:** Pituitary function, radiological and pathological findings and outcome in patients with fungal sinusitis with propagation to the sellar region (patients; n = 67).

Pituitary Function in Patients with Fungal Sinusitis with Propagation to the Sellar Region		
	n	%
Hypopituitarism	17	25.4
Hyperprolactinemia	9	13.4
Normal	10	14.9
Elevated ACTH	2	3.0
Acromegaly	1	1.5
AVP deficiency	1	1.5
No data	30	44.8
**Radiological and pathological findings**		
Pituitary abscess	28	41.8
Fungal granuloma	11	16.4
Aspergillosis	11	16.4
Allergic fungal sinusitis	10	14.9
Fungal sinusitis	4	6.0
Aspergillus infection	3	4.5
**Outcome of surgical and antifungal treatment**		
Recovery	45	67.2
Regression	6	9.0
Recurrence	2	3.0
Persisted blindness	1	1.5
Death	7	10.4
Unknown	6	9.0

ACTH—adrenocorticotropic hormone, AVP—arginine vasopressin.

**Table 3:** Serbian questionnaire for identification of patients with chronic sinusitis (www.fcf.org.rs).

Symptoms and Signs (S&S)	Present (Yes/No)	Duration of S&S
**Pain in head area**		
Head		
Face		
Ears		
Eye		
**Visual problems**		
Visual deterioration		
Ophthalmoplegia		
Visual field loss		
**Nasal problems**		
Congestion		
Runny nose		
Sneezing		
Cough		
Weakened sense of smell/taste		
**Sleeping and/or day activity problem**		
Lack of good sleep		
Waking up during the night		
Waking up tired		
Tiredness during the day		

## Data Availability

The original contributions presented in this study are included in the article. Further inquiries can be directed to the corresponding author.

## Abbreviations

The following abbreviations are used in this manuscript:

FS	Fungal sinusitis
AFS	Allergic fungal sinusitis
MRI	Magnetic resonance imaging
CT	Computed tomography
FT4	Free thyroxine
TSH	Thyroid-stimulating hormone
ACTH	Adrenocorticotropic hormone
IGF-1	Insulin-like growth factor 1
H&E	Hematoxilin and eosin
SIADH	Syndrome of inappropriate secretion of antidiuretic hormone
AVP	Arginine vasopressin
PCR	Polymerase chain reaction
ENT	Ear, nose, and throat doctor
